# Urban nature visitation, accessibility, and impact of travel distance for sustainable cities

**DOI:** 10.1038/s41598-023-44861-6

**Published:** 2023-10-18

**Authors:** Michelle L. Talal, Michal Gruntman

**Affiliations:** 1 Porter School of the Environment and Earth Sciences, Tel Aviv University, 6997801 Tel Aviv-Yafo, Israel; 2 School of Plant Sciences and Food Security, Tel Aviv University, 6997801 Tel Aviv-Yafo, Israel; 3 Present Address: Nicholas Institute for Energy, Environment & Sustainability, Duke University, Durham, NC 27708 USA

**Keywords:** Sustainability, Environmental sciences

## Abstract

Accessible urban nature is a key component of creating sustainable urban communities and promoting human health and well-being. To balance the economic, social, and environmental dimensions of sustainable development, the United Nations adopted several sustainable developmental goals (SDGs), such as SDG 11 for sustainable cities and communities, which aims to improve urban planning and management, including equitable access to urban nature. However, more information is still needed regarding how planners and managers can promote urban nature visitation and equitable access during health and environmental crises, such as the COVID-19 pandemic. The purpose of this study was to examine trends in urban nature visitation during the pandemic and then to determine if the effect of the pandemic on the frequency of urban nature site visitation varied by distance to home, using an innovative approach of analyzing both publicly available large-scale mobility data and a web-based survey of urban residents of Tel Aviv-Yafo, Israel. The mobility data results showed that there was a negative mean % difference in park visits compared to baseline during the first and third lockdowns, but an increase compared to baseline between lockdowns and even during the second lockdown. This suggests that urban residents had greater need to reconnect with urban nature during and after periods of intense stress. In addition, the survey results showed an increasing negative effect of distance on urban nature site visitation during the pandemic, specifically for urban nature sites located more than 1 km from home. Altogether, the mobility data and survey results suggest that people who lived within 1 km of their preferred urban nature site had disproportionate access to the benefits of urban nature during and after lockdowns than others. To effectively make social and ecological transitions toward urban sustainability, it is vital that cities promote urban nature accessibility during current and future environmental and health crises. Cities should collaborate with diverse stakeholders to create/maintain accessible urban nature sites nearby all sociodemographic groups, provide sustainability education and training to convey the benefits of urban nature, and pursue participatory solutions for understanding urban nature needs and preferences. In this manner, it will be possible to address the growing influence of proximity/travel distance and additional factors that affect urban nature visitation and ultimately, human health and urban sustainability.

## Introduction

As part of the United Nations’ effort to address the growth of human populations in cities and improve the quality of life of people, 193 countries adopted several sustainable development goals (SDGs) to balance the economic, social, and environmental dimensions of sustainable development^1^. To guide the transition toward a sustainable urban future, SDG 11 for sustainable cities and communities aims to improve urban planning and management in participatory and inclusive ways, such as promoting equitable access to urban nature in societies^1^. Urban nature provides a range of ecosystem services and contributes toward creating urban sustainability by reducing air/water pollution, conserving biodiversity, lowering air temperatures caused by the urban heat island effect, and promoting food sustainability^2–5^. In addition, visits to urban nature are associated with numerous health benefits for people such as decreased rates of stress, anxiety, obesity, and diabetes^6–9^ and are important for the promotion of sustainable lifestyles in cities even during crises^10^. For example, during the COVID-19 pandemic, there has been an increased public interest in urban nature and its many benefits^11–14^. However, more information is still needed regarding how planners and managers can promote urban nature visitation and equitable access during health and environmental crises^12,15–18^, especially since there has been a reduction in overall progress toward attaining SDGs during the COVID-19 pandemic^19,20^.

During the COVID-19 pandemic, urban nature has provided some of the most valuable and accessible places for the promotion of mental and physical health, particularly when many public places (e.g., gyms, schools, etc.) were closed due to various COVID-19-related lockdown restrictions^15,17,18,21–24^. People who visited urban nature more frequently during the pandemic tended to have lower levels of depression and stress, improved social cohesion, enhanced cognitive development, and increased rates of physical activity^23,25,26^. Previous studies examined the effect of the COVID-19 pandemic on visitation frequency and demand, showing either an increase^27–31^, decrease^25,32–34^, or relatively very little change for natural and semi-natural areas^35^. Some studies showed downward trends in visitation particularly during COVID-19-related lockdown periods^11,36^, with slight increases in park use after COVID-19-related restrictions were relaxed^34^.

There are several factors that may influence urban nature visitation frequency, and in particular, shorter travel distances to urban nature sites are often correlated with higher visitation frequencies^37–39^. Distance to urban nature is an important sustainability consideration in SDG 11’s aim of providing universal access to safe, inclusive and accessible, green and public spaces^1^. In addition, access to nearby urban nature can be helpful for meeting a range of other SDGs, such as SDG 4 for quality education (e.g., by reducing stress and improving cognitive development), SDG 7 for affordable and clean energy (e.g., by reducing nearby urban temperatures, energy bills, and use of carbon-emitted transportation methods), SDG 9 for industry, innovation, and infrastructure (e.g., by protecting local properties with stormwater management), and SDG 12 for responsible consumption and production (e.g., by providing nearby free and healthy food resources, such as fruit)^1–3,18,40–45^. Moreover, travel distance is likely to have become more influential for urban nature visitation during the COVID-19 pandemic due to various lockdown restrictions and/or concerns about virus transmission^17,36,46,47^. Only few studies, however, examined the role of distance in affecting these changes in urban nature visitation before versus during the COVID-19 pandemic, and their results revealed some different patterns. In some cases, people were willing and able to travel further distances to reach urban nature areas during the pandemic^15,28^, but others had a decrease in visitation to areas that were larger distances from home^15,29,35^.

To better understand these conflicting results, the purpose of this study is to investigate trends in urban nature visitation during the COVID-19 pandemic and to determine if the effect of the pandemic on the frequency of urban nature site visitation varied by distance to home. In this study, the following research questions were asked:

1. What are the trends of urban nature visitation during the COVID-19 pandemic?

2. How does distance to urban nature affect its visitation frequency during the COVID-19 pandemic?

To answer these questions, this study used an innovative combination of publicly available large-scale community mobility data and a web-based survey of urban residents in Tel Aviv-Yafo, Israel, regarding their urban nature visitation. There are some potential challenges in using big data and digital technologies in urban settings (e.g., privacy, equity, digital education/skills, digital disruptions, etc.), but they can enable cities to meet SDGs by providing opportunities for assessments, experimentation, innovation, and advancing toward sustainability targets^48,49^. Many previous studies tended to rely on mobility device location data to evaluate urban nature visitation^11,28,32,34^, but this study was unique in that it also incorporated a participatory approach with direct feedback in a web-based survey of urban residents.

In regards to the first research question, it was hypothesized that there would be downward trends in visitation during COVID-19-related lockdown periods in the city, which was shown in some previous studies of visitation trends using mobility data in other places such as Hungary^11^ and the Netherlands^36^. For the second research question, it was hypothesized that there would be less frequent visitation to urban nature sites with a greater distance from urban residents’ homes during the COVID-19 pandemic as compared to before, which was found in some previous research, including an international exploratory study^15^ and a case study in Finland^35^.

The findings of this type of urban nature study are important for making effective transitions toward urban sustainability and will help cities become more resilient during health and environmental crises. In accordance with SDG 11 for sustainable cities and communities, numerous countries are motivated to make cities more inclusive, safe, resilient, and sustainable. As a concept, sustainable cities and communities are strategic to supporting SDG 11, and new elements are needed to move forward toward SDGs. As important elements, planners and managers can better promote sustainable cities and communities by evaluating urban nature visitation trends and the potential influence of travel distance during crises. This information can be used toward protecting and/or restoring urban nature for equitable access across all sociodemographic groups. In addition to SDG 11, these efforts can help planners and managers pursue a range of other interdependent sustainability goals for a green transition, including SDG 4 (quality education), SDG 7 (affordable and clean energy), SDG 9 (industry, innovation, and infrastructure), and SDG 12 (responsible production and consumption)^1^. Altogether, nature in cities will need to be managed according to local climates and geographies, have adequate staffing and funding, and include community engagement to inform planning and strengthen human-nature connections^2,50,51^. In this manner, cities will be able to make progress toward SDGs by enhancing urban sustainability and providing inclusive and accessible urban nature.

## Materials and methods

### Study area

This study took place in Tel Aviv-Yafo, a city in Israel located on the Mediterranean coast with a human population of approximately 463,808 as of 2020^52^. This city aims to be a sustainable city by adopting an approach that incorporates several action areas, such as protecting and cultivating new urban nature and habitats, promoting a healthy life in a quality, no-pollution environment, and advancing sustainability education and training (i.e., both formal and informal educational frameworks) based on a multi-generational approach^53^.

**Figure 1:**
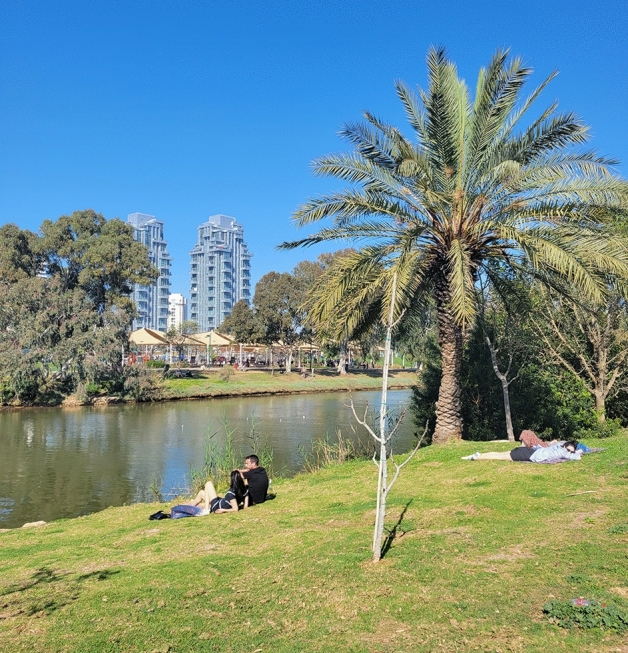
An urban nature site in Tel Aviv-Yafo, Israel, which includes gardens, physical recreation amenities, and the Yarkon River.

In Tel Aviv-Yafo, urban nature sites are defined as public lands within the city that are conserved, rehabilitated, and monitored to support nature and improve accessibility for people^53^. These sites include a variety of “green” and “blue” components such as gardens, beaches, parks, rivers, lakes, sand dunes, and green roofs that have been highlighted for their species diversity, ecological quality, and/or ecological connections to other sites in the city^53,54^. The urban nature sites are identified in the city’s master plan and the TA/5000 Zoning Plan for Tel Aviv-Yafo and assigned additional land use protections. An example of an urban nature site in Tel Aviv-Yafo is provided in Fig. 1.

In response to the COVID-19 pandemic, the Israeli government began enforcing public health measures in March 2020, followed by a series of COVID-19-related lockdown restrictions, such as stay-at-home orders, public spaces closures, and limitations on permitted travel distance from home^55^. There were three major pandemic-related lockdown periods in Israel that took place from March 19 to May 4, 2020 (Lockdown 1), September 18–October 18, 2020 (Lockdown 2), and December 27, 2020–February 7, 2021 (Lockdown 3), which each included a variety of rules such as stay-at-home orders, travel restrictions, social distancing, and closures of many public spaces^55,56^.

### Community mobility reports

To test the first research question, which was related to the trends of urban nature visitation during the COVID-19 pandemic, this study used the COVID-19 Community Mobility Reports of the “park” category for the Tel Aviv District^57^. The data show how visitation to (or time spent in) “parks” (defined as public gardens, castles, national forests, campgrounds, and observation decks) may have changed during the COVID-19 pandemic in comparison to baseline days determined from January 3 to February 6, 2020^57^. This information was used to calculate the average percent change from baseline for each day between March 19, 2020 (the day of the first lockdown) and May 5, 2021. Due to privacy considerations, this dataset does not publicly provide specific travel patterns or distances for individuals that enabled their location to Google LLC.

### Web-based survey

To test the second research question, which was regarding the effect of the COVID-19 pandemic on variations in the frequency of urban nature site visitation by distance to home, a web-based survey was developed in collaboration with local stakeholders and a focus group with urban residents in Tel Aviv-Yafo. The survey was distributed to participants living in Tel Aviv-Yafo from a range of demographic sectors by a local online survey panel company, Panel4All (http://www.panel4all.co.il) between March 21 and May 5, 2021 (Supplementary Material). The beginning of this timeframe was approximately 1.5 months after the end of a third lockdown in Israel. This web-based survey was part of a larger study that examined urban nature site visitation, as well as urban ecosystem services and nature preferences^58,59^. However, this present study was novel in that it was dedicated toward examining the potential influence of travel distance on urban nature site visitation during the COVID-19 pandemic in combination with large-scale community mobility data.

The survey asked participants to first select the urban nature site that they visited most often in Tel Aviv-Yafo, if applicable, and then approximately how often they visited this urban nature site before the COVID-19 pandemic (i.e., every day, 3–6 days/week, 1–2 days/week, every other week, once per month, every other month, once per year, or did not visit), during the COVID-19 pandemic. The survey also asked participates to indicate how far this urban nature site was from their home (i.e., scale of 0–15 km with 0.5 km intervals). To estimate each participant’s number of visits per year, the responses were recalculated in terms of the number of visits per year (e.g., once per month = 12 visits per year, 3–6 days per week = 234 visits per year, etc.). The distances from home were reclassified into five categories (e.g., 1 km or less, > 1–2 km, > 2–5 km, > 5–10 km, and > 10–15 km). The optimal sample size for the survey was 384 participants, which was based on the population size of Tel Aviv-Yafo with a 5% margin of error and a 95% confidence level^60,61^. The sampling effort was limited by funding considerations, but sampling was continued until the optimal same size was reached with an adequate demographic representation.

### Statistical analyses

The COVID-19 Community Mobility Reports^57^ data were analyzed with analysis of variance (ANOVA) to determine if there were downward trends in visitation during COVID-19-related lockdown periods in the city. The percent change from baseline per day was used as the dependent variable, timing (i.e., during lockdown or after lockdown) was used as a fixed factor, and lockdown number in Tel Aviv-Yafo (i.e., Lockdown 1, 2, or 3) was the random factor.

The data from the web-based survey were analyzed using a repeated-measures ANOVA. The urban nature site visitation frequency per year was used as the dependent variable, timing (i.e., before or during the COVID-19 pandemic) was used as the within-subject factor, and distance (km) of the urban nature sites to participants’ homes was used as the between-subject factor. A log(x + 1) transformation was performed on the dependent variable to meet the assumption of a normal distribution before conducting the repeated-measures ANOVA. All analyses were conducted with IBM SPSS, Version 28.

### Institutional review board statement

The web-based survey was approved by the Tel Aviv University Institutional Review Board (IRB) on February 2, 2021 (No. 0002670-1). All methods were performed in accordance with relevant guidelines and regulations, including in accordance with the Declaration of Helsinki. Each of the participants and/or their legal guardians provided informed written consent for their study participation and publication, and were at least 18 years old. Any and all identifying information about the participants was removed from the manuscript and supplementary material.

## Results

### Community mobility reports

**Figure 2:**
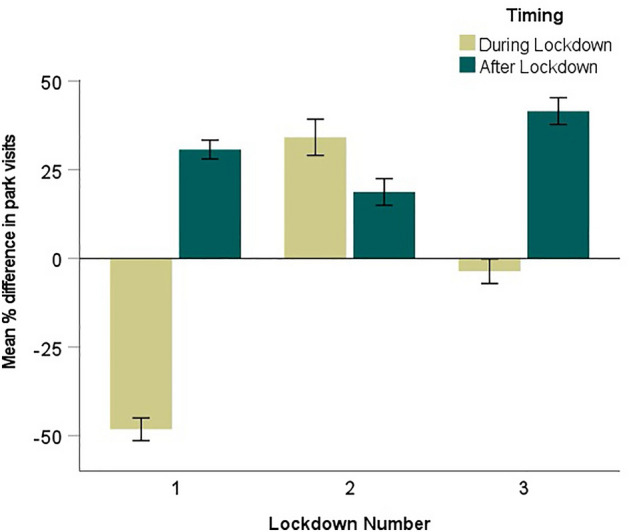
Mean % difference in park visits (± 1 SE) in the Tel Aviv District from the COVID-19 Community Mobility Report^57^ baseline of January 3–February 6, 2020, showing during and after each of the three COVID-19 related lockdowns in Israel.

During the period of February 2020–May 2021, the COVID-19 Community Mobility Reports for the Tel Aviv District^57^ data showed an average of an 18% increase in visitation compared to the January 3–February 6, 2020 baseline. This increase was shown mainly in the periods between/following lockdowns, with the highest mean % increase in park visits compared to the baseline occurring after the third lockdown (+ 42%; Fig. 2). There was a decrease in mean % difference in park visits compared to baseline during the first and third lockdowns, with the largest decline during the first lockdown (-48%). However, the ANOVA results indicated that the mean % difference in park visits was not affected solely by lockdown number (F_2,408_ = 0.648, p = 0.607) or timing (i.e., during or after lockdown: F_1,408_ = 1.801, p = 0.312), but rather by both factors (Timing × Lockdown Number: F_2,408_ = 63.22, p < 0.001). This interactive effect is mainly due to the increase in park visits shown during the second lockdown as compared to afterwards.

### Web-based survey

A total of 472 residents of Tel Aviv-Yafo participated in the survey. Of the surveys, 14 were removed due to inconsistent and/or incomplete data. The demographics of the remaining study participants (n = 458) were relatively comparable to that of the Tel Aviv-Yafo region^62^, except for a slightly higher percentage of female participants, slightly higher median age, and slightly lower percentage of underrepresented religious/ethnic groups (Table 1). Some of the discrepancies between the demographics may be because the last full census for the Tel Aviv-Yafo region was completed in 2008. In addition, some of the demographic variables in the census included individuals who were 15 years old or older, whereas the web-based survey only included participants who were at least 18 years old.

**Table 1:** Demographics information for Tel Aviv-Yafo, Israel, and all participants.

Demographic	Gender	Age	Education	Religion/Ethnicity
Tel Aviv-Yafo Region^a^	51% Female 49% Male	34 (median)	12% < High school completion^b^ 26% High school^b^ 23% Bachelor’s degree^b^ 14% Second academic degree or higher^b^	0.7% Christian 0.0% Druze 93.7% Jewish 1.0% Muslim 4.5% Other
Participants	56% Female 44% Male	18–91 (range), 38 (median)	2% < High school completion 24% High school 12% Partial college 36% Bachelor’s degree 24% Master’s degree 2% PhD	0.22% Christian 0.44% Druze 98.9% Jewish 0.22% Muslim 0.44% Other

^a^Data from the 2008 Census of Population^62^.

^b^Percentage includes individuals at least 15 years old.

**Figure 3:**
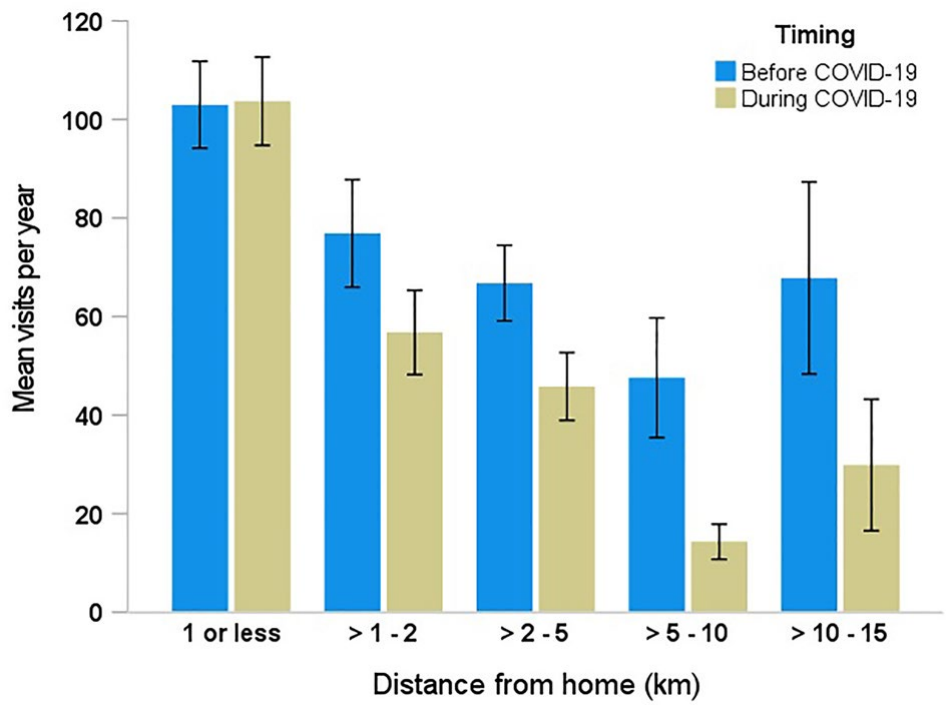
Urban nature site visitation frequency (mean visits per year ± 1 SE) by distance (km) before and during the COVID-19 pandemic.

The web-based survey indicated that a total of 421 participants (92% of the 458 participants) visited urban nature sites at least once before or during the COVID-19 pandemic. Most visitors (85%) indicated that the urban nature site they visited most often was located 5 km or less from their home, while only 15% selected one that was located more than 5 km from their home. The repeated-measures ANOVA indicated that urban nature site visit frequency was negatively affected by both distance from home (F_4,420_ = 12.348, p < 0.001) and the COVID-19 pandemic (F_1,420_ = 41.734, p < 0.001) (Fig. 3). The negative effect of distance, however, was further exacerbated during the COVID-19 pandemic (Timing × Distance: F_4,420_ = 3.983, p = 0.003), indicating a more intense decrease in urban nature visitation during the COVID-19 pandemic to sites with greater distances from home as compared to before the pandemic.

## Discussion

Urban nature visitation and accessibility are important for promoting sustainable cities and equitable access to urban nature in societies during environmental and health crises^11,17^. Understanding urban native visitation trends and the potential influence of travel distance can help cities make transitions toward sustainability in accordance with United Nations’ SDG 11 for sustainable cities and communities^1^, as well as a range of other intersecting goals, such as SDG 4 (quality education), SDG 7 (affordable and clean energy), SDG 9 (industry, innovation, and infrastructure), and SDG 12 (responsible production and consumption)^1^. To examine general trends in urban nature usage and the effect of distance on urban nature site visitation before and during the COVID-19 pandemic, this study used an innovative combination of both publicly available community mobility data^57^ and a web-based survey of urban residents, in a case study of Tel Aviv-Yafo, Israel. This type of information is important for countries that are dedicated toward making their cities more sustainable and resilient by promoting the protection/cultivation of urban nature and a healthy quality of life for people^1,63^.

The Community Mobility Report data for the Tel Aviv District^57^ revealed trends in urban nature visitation during and after three major lockdowns in the city. It was hypothesized that there would be decreasing trends in urban nature visitation during each of the lockdowns, based on the findings of previous studies in the Netherlands^36^, Israel^33^, the United States^64^, and Hungary^11^. However, in the case of Tel Aviv-Yafo, the data indicated that there was a negative mean % difference in park visits during the first and third lockdowns, but not during the second lockdown, which had an increase in visits compared to the baseline. It is possible that this large increase in park visitation during the second lockdown resulted from the stress incurred during the first lockdown, when residents experienced the largest decrease in visitation. In general, this study showed increasing trends in park visitation after each lockdown, which was similar to a study in the United States^34^. Altogether, the data suggests that urban residents had a great need to reconnect with urban nature after periods of intense stress and had resilience during various stages of the pandemic^22,35^.

This study also investigated if there was an effect of the COVID-19 pandemic on the frequency of urban nature site visitation with varying distance from home. Consistent with the hypothesis, the results showed that there was less frequent visitation to urban nature sites in Tel Aviv-Yafo that were a greater distance from home during as compared to before the pandemic. The web-based survey indicated that urban nature site visitation declined for most of the participants who lived more than 1 km from their preferred urban nature site. However, there was not a similar decline in visitation frequency for participants who lived 1 km or less from their preferred urban nature site. These findings are different from studies that showed that some individuals were willing and able to travel further distances during the pandemic to reach urban nature^15,28^. On the other hand, the results of this study are consistent with other research in which participants described the importance of having accessible urban nature near their homes during the COVID-19 pandemic^15,66^. Combined with the results of community mobility data, these findings suggest that urban residents who lived 1 km or less from their preferred urban nature site most likely experienced enhanced privilege in accessing the benefits of urban nature during and after lockdowns (e.g., particularly during the second lockdown when mobility was limited), as compared to people who lived more than 1 km away.

The results of this study have important urban planning and management implications for making transitions toward sustainable cities and communities in alignment with SDG 11^1^. Investigating urban nature visitation trends and the potential influence of travel distance during crises are important elements to move forward toward SDGs and support sustainable cities and communities. In the case of Tel Aviv-Yafo, there was less urban nature visitation before and especially during the COVID-19 pandemic for participants who lived more than 1 km from their preferred sites. To ensure that there is equitable access to the numerous benefits of urban nature (e.g., recreational opportunities, cleaner air, lower air temperatures, etc.)^6–8^, urban nature sites should be created and maintained near home for all urban residents. It is important for residents to have nearby urban nature that is walkable and/or bikeable, particularly during health and/or environmental crises when other forms of transportation (e.g., buses, trains, etc.) may not be available and/or may be riskier for contracting viruses or other illnesses^17,36^. In this manner, cities will be able to plan for a more sustainable future, one in which residents will be able to access urban nature with minimal travel distances, thereby also reducing the need for carbon-emitting public and/or private transportation. This is especially important in times of crises when some groups (e.g., underrepresented racial/ethnic communities, families with young children, older individuals, lower income groups, etc.) may be more vulnerable than others to the negative effects of restrictions such as lockdowns and limited travel distances^21,65^.

Urban planners and managers can enhance urban sustainability by cultivating accessible nature across urban landscapes, particularly in underserved neighborhoods, while also minimizing green gentrification^67,68^. In addition to proximity of urban nature sites, planners and managers should incorporate several other important accessibility considerations for urban nature, such as amenities, size, maintenance, multi-sensory experiences, educational materials for cultural and natural resources (e.g., on-site and digital in multiple languages, braille, etc.), as well as increased safety and security for women, children, underrepresented racial/ethnic groups, elderly, and people with mental and/or physical health conditions^18,71^. In addition, urban planners and managers should pursue participatory solutions with diverse stakeholders to learn more about accessibility needs and opportunities for urban nature^21,73^. In this manner, they will be better prepared to balance both social and ecological goals for urban nature management^74,75^. Overall, these accessibility improvements are also likely to help communities become more resilient and sustainable during current and potential future health and/or environmental crises^12,13^.

To make progress toward urban sustainability goals, it is also necessary that cities provide formal and informal educational opportunities to diverse stakeholders on environmental issues that are critical for sustainable development (e.g., biodiversity, climate change, accessibility, energy consumption, etc.)^3,78^. For example, education for sustainable development (ESD) is a global initiative that is focused on achieving SDGs through providing people with the knowledge, skills, values, and attitudes to make contributions to sustainable development in their communities^79^. This type of initiative not only contributes to SDG 4 (quality education), but is strongly interconnected with several other SDGs that address complex issues related to sustainable cities^76,79^. In addition, as societies continue to undergo digital transformations and integrate new technologies, it is important that educators use a variety technology-assisted learning tools (e.g., mobile devices, laptops, virtual laboratories, interactive games, etc.) to improve learning experiences and prepare students to use modern sustainability technologies (e.g., technology aiding efficient energy consumption, reducing carbon emissions)^49,80^. Beyond the use of digital technologies, sustainability education should include real-world, meaningful experiences in urban nature that promote strong human-nature relationships^81^. Moreover, these nature experiences are also likely to improve education by reducing anxiety and stress, increasing concentration, enhancing student performance, and promoting sustainable actions (e.g., lifestyles, ecological behavior, etc.)^2,41^.

### Limitations and future research

The results of this study suggest that using large-scale community mobility data for identifying variations in visitation frequency before and during the COVID-19 pandemic might be less reliable, but that they can still be advantageous for examining shorter-term visitation trends in relation to COVID-19-related lockdown periods, as well as monitoring and forecasting trends during disaster events such as pandemics^82^. For example, the community mobility data^57^ showed increases and decreases in park visitation at different times of the COVID-19 pandemic, but an overall 18% increase in visitation during the pandemic compared to the baseline. This result contrasted with the results of the web-based survey and another study of urban nature visitation in Tel Aviv-Yafo, which showed a decrease in visitation during the pandemic^33^. This inconsistency for overall visitation between the data sources may be due to a key limitation of the community mobility data, specifically that its baseline days were taken during wintertime in Tel Aviv-Yafo. This likely created a bias toward increased mobility during later months with warmer temperatures and better weather conditions for visiting urban nature.

The COVID-19 Community Mobility Reports^57^ may also be limited in that it only included data that were collected from individuals who enabled their location information on their mobile phones to Google LLC. The dataset did not distinguish travel distances for individuals or trends for different types of nature in the city, but rather combined them together as “parks” (i.e., public gardens, castles, national forests, campgrounds, and observation decks). Google LLC (2022) also advises caution in using this data for long-term analysis, as populations and behavior may have greatly shifted compared to the original baseline period. For these reasons, an alternate baseline period or a web-based survey may be more valuable in future research for gathering information about overall visitation frequency before and during a health and/or environmental crisis. In addition, future studies would benefit from having greater differentiation of the types of nature in the city so that researchers can better understand use patterns. Future advancements in urban big data and digital technologies are likely to help cities assess urban nature use and accessibility, as well as advance toward sustainability goals^48,49^.

The web-based survey of residents in Tel Aviv-Yafo may have some limitations in that it was distributed during a particular time period one year into the pandemic, which may have limited the participants’ accurate recollection of their visitation frequency to urban nature sites prior to the pandemic. The visitation behavior of the participants may have also shifted during different parts of the pandemic, including during various levels of lockdown-related restrictions. It is possible that participants visited more than one designated urban nature site before and/or during the pandemic, as well as other types of urban nature (e.g., backyards, parks, community gardens, etc.), but participants were only asked to select one preferred urban nature site to better compare visitation and distance to home for each participant. To protect anonymity, participants were not asked to provide their home addresses, but rather asked to estimate how far their preferred site was from home. It is possible that there were some approximation errors, but these errors were not likely to differ between estimations provided by the same participant before and during the pandemic. Future studies should include additional questions for participants regarding their specific behavior during different times of the pandemic (e.g., before, during, and after lockdowns), relate to multiple types of urban nature, and ask more in-depth questions regarding urban nature accessibility and sustainability during crises.

## Conclusions

Urban nature has provided numerous physical and mental health benefits to people before and during the COVID-19 pandemic and is essential for meeting the United Nations SDG 11 for sustainable cities and communities, as well as a range of other SDGs^1^. This study of Tel Aviv-Yafo, Israel, examined general trends in urban nature usage and the effect of distance on urban nature site visitation before and during the COVID-19 pandemic, using a unique combination of both publicly available community mobility data^57^ and a web-based survey of urban residents. Altogether, the combined results of the mobility data and web-based survey suggest that people who lived 1 km or less from their preferred urban nature site had increased privilege in accessing urban nature and its many benefits during and after lockdowns (e.g., and particularly during the second lockdown) compared to urban residents who lived more than 1 km away. This emphasizes the need for more equitable access to urban nature, especially during times of crises and lockdowns when mobility is limited and urban nature can provide mental and health benefits for people under stress.

To effectively make social and ecological transitions toward urban sustainability, it is important that cities promote urban nature accessibility during current and future environmental and health crises, such as the COVID-19 pandemic, global climate change, and rapid land use change and development. As nations continue to adaptively manage their responses to the COVID-19 pandemic and other health and environmental emergencies, environmental decision-makers should collaborate with diverse stakeholders to create and maintain urban nature sites near all urban residents across the urban landscape. In this way, urban nature will be more accessible particularly during crises when other forms of transportation may not be safe or available. Cities should continue to convey the benefits of urban nature through sustainability education and training in both formal and informal settings that are inclusive to underrepresented and underserved sociodemographic groups. In addition, they should pursue participatory solutions for understanding the urban nature accessibility needs and preferences of diverse stakeholders. In this manner, it will be possible to address the growing influence of proximity/travel distance and additional factors that affect urban nature visitation and ultimately, human health and sustainability in cities.

### Supplementary Information

Supplementary Information.

## Data Availability

Data used in the analysis are provided at 10.5281/zenodo.5807737. Please contact the corresponding author (MLT) for additional information.
