# Supplementary material for: Urban nature visitation, accessibility, and impact of travel distance for sustainable cities
Source: Sci Rep. 2023 Oct 18;13:17808. doi: 10.1038/s41598-023-44861-6 (PMC10584958; doi:10.1038/s41598-023-44861-6)
Supplement: Supplementary file 1 — Supplementary Information. [file 41598_2023_44861_MOESM1_ESM.pdf]

••• Academic Secretariat
Senate Office

TEL AVIV UNIVERSITY אוניברסיטת תל אביב

••• המזכירות האקדמית
מדור הסנאט

February 15, 2021

Dr. Michal Gruntman
Faculty of Life Sciences
Tel Aviv University
Tel Aviv, 69978, Israel

This is to inform you that your research proposal no. 0002670-1 entitled:

"Urban nature site ecosystem services, impacts on quality of life, and nature preferences"

meets the requirements of the Ethics Committee of Tel-Aviv University.

The investigator's information:

The document is for ethical purposes only and valid until 14-02-2022.

*** Please note: Applications for the extension of a certificate will be submitted one month before the approval expires.**

Sincerely,

בן ענת מנחם.

Prof. Meir Lahav
Chairman, The Ethics Committee
Tel-Aviv University

TEL-AVIV UNIVERSITY
THE ETHICS COMMITTEE

Urban Nature Site Visitors – Survey

Purpose: The purpose of this study is to examine urban nature site visitor preferences, perceptions of benefits, and contribution to quality of life in general and during the COVID-19 (Corona) pandemic. Urban nature sites are public spaces in the city that are conserved, rehabilitated, and monitored to support nature and provide enjoyment for people. The results of this study may be used by planners and managers to improve urban nature sites.

Activities: Participants will be asked a series of questions about their preferences and perceptions, as well as some demographics questions.

Confidentiality: The survey is anonymous and no information will be collected regarding the subjects' identity. Unidentifiable data will be shared among the study's researchers.

Voluntariness: Participation in the study is voluntary and there is no penalty for choosing to not participate in the study or leave at any time.

Contact Information:

Dr. Michelle Talal, Tel Aviv University, michelleta@tauex.tau.ac.il
Dr. Michal Gruntman, Tel Aviv University
Dr. Shiri Zemah Shamir, IDC Herzliya

Sponsor: Tel Aviv University, Zuckerman STEM Leadership Program

Do you consent to completing this survey? You must be at least 18 years old to give consent.

- ☐ Yes
- ☐ No

(Note – if participant does not consent, then they will not qualify to take survey)

1. **Select the ONE (1) urban nature site that you visit most often in Tel Aviv-Yafo.**

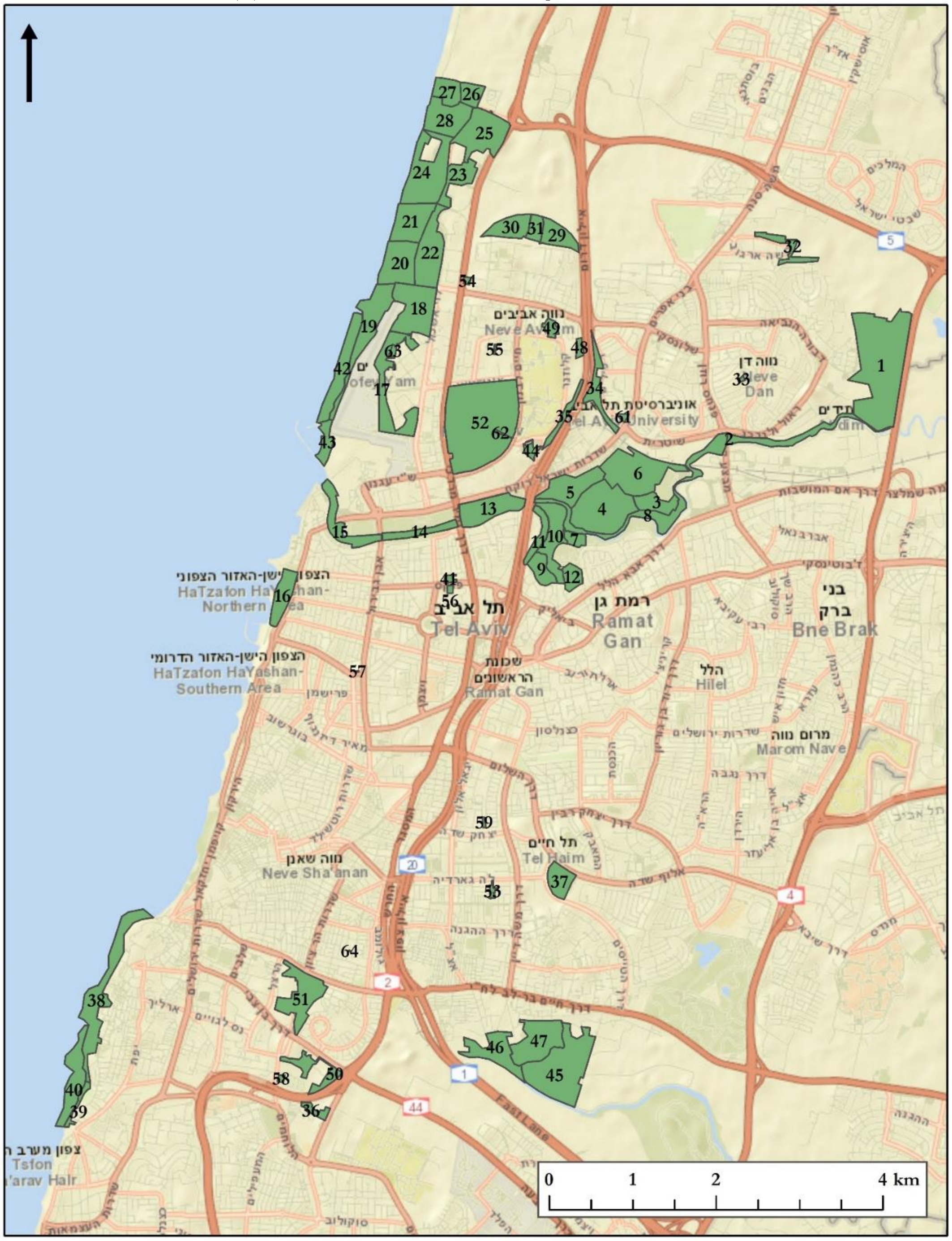

(The authors created the figure using ArcGIS Desktop 10.8.1, https://www.esri.com/en-us/arcgis/products/arcgis-desktop/overview)

1	נחל פרדסים	33	תל נוריה
2	פארק ירקון מזרח	34	רפידים
3	פארק ירקון בור	35	מורדות האוניברסיטה
4	פארק ירקון אגם	36	בריכת הלוחמים חולון
5	פארק ירקון חורשה צפונית	37	פארק אדית וולפסון
6	פארק ירקון מדשאה אגם	38	מדרון יפו
7	מתחם שבע טחנות	41	גן יד לבנים
8	נחל ירקון מקטע האגם	45	פארק בגין מזרח
9	חורשת ראש ציפור	46	פארק בגין מערב
10	ראש ציפור	47	פארק בגין אגם
11	נחל איילון	49	גן קרטר
12	גבעת נפוליאון	51	פארק החורשות
13	נחל ירקון מקטע בבלי	48	חורשת בון
14	נחל ירקון מקטע ספורטק	39	חוף גבעת עליה וגן יצחק שדה
15	נחל ירקון מקטע מערבי	44	שיח מוניס
16	גן העצמאות	50	תל גיבורים
17	שדה דב דרום	40	ים גבעת עליה
18	שדה דב צפון	43	חוף רידינג
19	חוף תל ברוך	42	ים תל ברוך
20	רקית דרום	52	רמת אביב א'
21	תל רקית	53	חורשת יגור
22	רקית מזרח	54	בויאר
23	בריכת לוינסקי דרום	55	סמטת טאגור
24	הצוק דרום	56	אהבת ציון
25	בריכת לוינסקי צפון	57	גג בניין העירייה
26	קאנטרי קלאב גלילות	58	
27	הצוק צפון	59	שדרות ההשכלה
28	הצוק מנדרין	60	יד לבנים (פנקס / בני משה)
29	אפקה מזרח	61	רפידים / בני אפרים
30	אפקה מערב	62	שדרות אבנר (ברודצקי / לבנון)
31	אפקה מרכז	63	נופי ים
32	חורשת אבנר צהלה	64	קפה שפירא

☐ I do not visit any of these urban nature sites.
(Note – participant will not be told upfront, but this answer will skip to Question 17)

2. Approximately how often did you visit this urban nature site <u>before</u> the COVID-19 (Corona) pandemic began?

☐ Every day
☐ 4 – 6 days per week (combined with next option because of the overlap: 3-6 days)
☐ 3 – 5 days per week
☐ 1 – 2 days per week
☐ Every other week
☐ 1 time per month
☐ Every other month
☐ 1 time per year
☐ I did not visit

3. **Approximately how often did you visit this urban nature site <u>during</u> the COVID-19 (Corona) pandemic?**
   - ☐ Every day
   - ☐ 4 – 6 days per week (combined with next option because of the overlap: 3-6 days)
   - ☐ 3 – 5 days per week
   - ☐ 1 – 2 days per week
   - ☐ Every other week
   - ☐ 1 time per month
   - ☐ Every other month
   - ☐ 1 time per year
   - ☐ I did not visit

4. **Do you usually visit this urban nature site (check all that apply):**
   - ☐ Alone
   - ☐ With friends
   - ☐ With family
   - ☐ With children
   - ☐ With a date / romantic partner
   - ☐ With a dog(s)

5. **Approximately how far is this urban nature site from your home?**

   (Insert scale from 0 to 15 km, with 0.5 km intervals).

6. **How do you travel to this urban nature site (select all that apply)?**
   - ☐ Bike
   - ☐ Bus
   - ☐ Car
   - ☐ Motorcycle
   - ☐ Scooter
   - ☐ Skateboard
   - ☐ Walk
   - ☐ Wheelchair
   - ☐ Other: Write-in

7. **Approximately how long do you normally stay in the urban nature site per visit?**
   - ☐ Less than 1 hour
   - ☐ 1 hour
   - ☐ 2 hours
   - ☐ 3 hours
   - ☐ 4 hours

- ☐ 5 – 8 hours
- ☐ More than 8 hours

8. If there was an entrance fee to this urban nature site, how much would you be willing to pay per visit?

- ☐ 0 NIS
- ☐ 5 NIS
- ☐ 10 NIS
- ☐ 15 NIS
- ☐ 20 NIS
- ☐ 25 NIS
- ☐ 30 NIS
- ☐ 35 NIS
- ☐ 40 NIS
- ☐ 45 NIS
- ☐ 50 NIS
- ☐ Other: Write-in

9. Is this urban nature site easily accessible to you?

Strongly Disagree	Disagree	Undecided	Agree	Strongly Agree
O	O	O	O	O

10. In general, how would you rate your visits to this urban nature site?

Very Poor	Poor	Fair	Good	Excellent
O	O	O	O	O

11. Why do you visit this urban nature site (select all that apply)?

- ☐ Beauty
- ☐ Biking
- ☐ Dating
- ☐ Dog walking
- ☐ Educational activities
- ☐ Nature experience
- ☐ Picnic
- ☐ Photography
- ☐ Proximity to home or work
- ☐ Recreational fishing
- ☐ Relaxation
- ☐ Running

- ☐ Solitude
- ☐ Socialize with children
- ☐ Socialize with family
- ☐ Socialize with friends
- ☐ Walking
- ☐ Work
- ☐ Other: Write-in

12. Before the COVID-19 (Corona) pandemic began, how much did your visits to this urban nature site contribute toward your:

	Never	Rarely	Sometimes	Often	Always	I did not visit before the COVID-19 (Corona) pandemic
Physical health?	O	O	O	O	O	O
Mental health?	O	O	O	O	O	O

13. During the past 12-months of the COVID-19 (Corona) pandemic, how much have your visits to this urban nature site contributed toward your:

	Never	Rarely	Sometimes	Often	Always	I did not visit during the COVID-19 (Corona) pandemic
Physical health?	O	O	O	O	O	O
Mental health?	O	O	O	O	O	O

14. In your opinion, how would you rate this urban nature site's ability to:

	Very Poor	Poor	Fair	Good	Excellent
Provide physical recreational opportunities (e.g., biking, walking, etc.)?	O	O	O	O	O
Provide relaxation opportunities (e.g., sitting, socializing, solitude, etc.)?	O	O	O	O	O
Provide beauty?	O	O	O	O	O
Provide educational opportunities (e.g., informational signs, studying)?	O	O	O	O	O
Provide food collection opportunities (e.g., for fruit, herbs, etc.)?	O	O	O	O	O
Clean the air?	O	O	O	O	O
Reduce city noise?	O	O	O	O	O
Lower the air temperature?	O	O	O	O	O
Purify water?	O	O	O	O	O
Provide nature in the city?	O	O	O	O	O

Provide habitat for plants?	O	O	O	O	O
Provide habitat for animals?	O	O	O	O	O
Support biodiversity?	O	O	O	O	O

15. In this urban nature site, how would you rate:

	Very Poor	Poor	Fair	Good	Excellent
The maintenance of the human-made structures?	O	O	O	O	O
The maintenance of the nature?	O	O	O	O	O
Safety?	O	O	O	O	O

16. What improvements, if any, do you recommend for this urban nature site? Write-in

17. In general (not specific to a particular site), what are the FIVE (5) most important characteristics to have in an urban nature site?

Maintenance of human-made structures	Quiet atmosphere	Relaxation opportunities (e.g., sitting, socializing, solitude, etc.).	Plant habitat
Maintenance of nature	Lower air temperature	Beauty	Animal habitat
Safety	Water purification	Educational opportunities (e.g., informational signs, studying)	Biodiversity
Clean air	Physical recreation opportunities (e.g., biking, walking, etc.)	Nature in the city	Food collection opportunities (e.g., for fruits, herbs, etc.)

- ☐ No opinion
- ☐ None of these
- ☐ Other: Write-in

18. In general (not specific to a site), how important is it to you to have these components in an urban nature site?

	Not Important	Slightly Important	Moderately Important	Very Important	Extremely Important
Spring	O	O	O	O	O
Pond	O	O	O	O	O
Lake	O	O	O	O	O
Stream	O	O	O	O	O
Sea	O	O	O	O	O
Shrubs	O	O	O	O	O
Trees	O	O	O	O	O
Grasses	O	O	O	O	O
Flowers	O	O	O	O	O
Birds	O	O	O	O	O

Invertebrates	O	O	O	O	O
Mammals	O	O	O	O	O
Reptiles	O	O	O	O	O
Amphibians	O	O	O	O	O
Fish	O	O	O	O	O
Sand	O	O	O	O	O
Bare soil	O	O	O	O	O
Caves	O	O	O	O	O
Hills	O	O	O	O	O
Large rocks	O	O	O	O	O

19. Which are important for the future of urban nature sites in the city (select all that apply)?

☐ Create new urban nature sites.

☐ Improve existing urban nature sites.

☐ Increase connections between urban nature sites.

☐ Reduce the number of urban nature sites.

☐ No changes are needed.

☐ Other: Write-in

20. Please indicate for each of the following 5 statements which is the closest to how you have been feeling over the past 2 weeks:

	All of the time	Most of the time	More than half the time	Less than half of the time	Some of the time	At no time
I have felt cheerful and in good spirits.	O	O	O	O	O	O
I have felt calm and relaxed.	O	O	O	O	O	O
I have felt active and vigorous.	O	O	O	O	O	O
I woke up feeling fresh and rested.	O	O	O	O	O	O
My daily life has been filled with things that interest me.	O	O	O	O	O	O

21. Age (years): Write-in

22. Gender:

☐ Female

☐ Male

☐ Other: Write-in

23. Religion/Ethnicity (please check all that apply):
- ☐ Jewish
- ☐ Muslim
- ☐ Christian
- ☐ Druze
- ☐ Other: Write-in

24. Where did you primarily grow up between birth – 18 years old (city/town and country)? Write-in

25. Which best defines you?
- ☐ Resident
- ☐ Tourist
- ☐ International Student/Worker

26. Do you live:
- ☐ Alone
- ☐ With friends / roommate(s)
- ☐ With family

27. Education Level:
- ☐ Less than high school degree
- ☐ High school degree or equivalent
- ☐ Some college but no degree
- ☐ Bachelor's degree
- ☐ Master's degree
- ☐ Doctorate degree

28. Education Field of Study (Write N/A if not applicable): Write-in

29. Employment Status before the COVID-19 (Corona) pandemic:
- ☐ Full-time
- ☐ Part-time
- ☐ Unemployed
- ☐ Retired
- ☐ Soldier
- ☐ Student

30. Employment Status during the COVID-19 (Corona) pandemic:
- ☐ Full-time
- ☐ Part-time
- ☐ Unemployed

- ☐ Retired
- ☐ Soldier
- ☐ Student

31. Please use the space below to write any comments you have about this survey or our research. Write-in
